# Genetic Diversity Based on the Analysis of 27 Y- Short Tandem Repetition (STR) Loci in Two Populations in the Apuseni Mountains, Romania

**DOI:** 10.7759/cureus.62505

**Published:** 2024-06-17

**Authors:** Ramona Hodișan, Dana C Zaha, Claudia M Jurca, Streba Irina, Marius Bembea

**Affiliations:** 1 Doctoral School of Biomedical Sciences, University of Oradea, Oradea, ROU; 2 Department of Preclinical Disciplines, University of Oradea, Faculty of Medicine and Pharmacy, Oradea, ROU; 3 Institute of Forensic Medicine, Iasi, Iasi, ROU

**Keywords:** human, romania population, haplogroup, haplotype diversity, genetic diversity, y-chromosome, y-str

## Abstract

Background: Y chromosome analysis is used in various fields of forensic genetics, genetic genealogy, and evolutionary research, due to its unique characteristics. Short tandem repetitions (STR) are particularly relevant in population genetic studies. The aim of this study is to analyze the genetic profile of two populations in the Apuseni Mountains area, Băița and Roșia Montană, Romania.

Methods: 27 STR loci of the Y chromosome were analyzed to investigate the genetic profile of two populations from the Apuseni Mountains area. Investigating genetic diversity by analyzing allele frequency, haplotype frequency, calculating forensic parameters, and presenting the main haplogroups identified based on Y-STR markers.

Results: Gene diversity in the batch from Băița varies from 0.515 for the DYS393 locus to 0.947 for the DYS385 locus. In the Roșia Montană population, gene diversity ranges from 0.432 for DYS393 to 0.931 for DYS385. The haplotype diversity in Roșia Montană was 0.991, and the haplotype diversity was 1.000 in the population from Băița. A total of nine haplogroups was identified in the batch from Băița, while only seven haplogroups were observed in the batch from Roșia Montană. Both groups are based on the same five major haplogroups (E, G, I, J, and R) and the most common haplogroup is R1b in both populations.

Conclusion: In this study, the genetic diversity of two distinct populations was assessed using genetic analyses based on different markers. Analysis of Y-STR profiles revealed significant genetic diversity in both studied groups. All haplogroups identified were similar to those present in other Romanian populations.

## Introduction

The Y chromosome, with a relatively small size of about 60 Mb, is transmitted from father to son without significant changes, except for occasional mutations. Approximately 50% of the Y chromosome consists of repetitive sequences, such as single-base substitutions, Alu elements, and long intercalated nuclear elements. Among these, short tandem repetitions (STRs) are relevant in genetic studies of populations. They have an average frequency of mutation of about 0.2% per generation [1].

Y chromosome analysis is widely used in various fields of forensic genetics, genetic genealogy, and evolutionary research due to its distinctive characteristics [2]. Due to their pattern of paternal inheritance, Y-STRs are valuable in family genealogy investigations, ancestral origin analysis, and the identification of male/female DNA mixtures. These characteristics make Y-STRs important tools in historical and genealogical study, as well as in the field of forensics [3].

Y chromosome haplogroups can be successfully predicted from Y-STR markers using Y-STR haplogroup prediction tools. This method has recently attracted attention due to its efficiency in terms of labor, time, and associated costs. The Y-STR haplotype represents the set of alleles on the same chromosome. At the same time, major haplogroups (branches of the Y chromosome phylogeny) reflect the establishment and expansion of major population groups and can provide information about the time scale and route of major migration events [1].

Roșia Montană, located in Alba County, and Băița, part of the town of Nucet in Bihor County, are two mining settlements located at a road distance of about 80 km in the Apuseni Mountains region of Transylvania, Romania. According to the Population and Housing Census of 2021, Roșia Montană has a population of 2.428, of whom 74.05% are Romanian, while 16.27% are from other ethnic groups. In contrast, the town of Nucet has 1987 inhabitants, with 89.03% of them being of Romanian ethnicity [4].

Roșia Montană commune is located in the northeastern part of the Metalliferous Mountains and the “golden polygon”, 75 km from Alba Iulia, the county residence of Alba, and 10 respectively, 12 km, from the towns of Abrud and Câmpeni. The commune consists of 16 villages spread over an area of 5168 hectares [5].

Roșia Montană is a mining settlement and an example of the coexistence of a great diversity of ethnicities and religions throughout history. The first exploitation activities are attributed by archaeologists to the Neolithic civilization they continued in the Bronze Age, simultaneously with the development of the art of gold processing in the geographical space of today’s Transylvania [6].

Starting with the discovery of four wooden boards in 1786 in the gallery of a mine, the Roman exploitation of the gold mines at Roșia Montană was officially attested. The number of these tablets increased to over 25 by 1855, representing contractual documents dating back to the middle of the second century AD. These artifacts confirmed the presence of mining communities in the area and provided a fascinating insight into the mining activity of that period. The first documented mention of the village dates back to the Roman period, under the name of Alburnus Maior [7].

In the area of the Carpathian arc, military attacks followed centuries after the Aurelian withdrawal, including invasions by Ostrogoths, Visigoths, Huns, Avars, Slavs, and ultimately Hungarians in Transylvania. However, as far as Roșia Montană is concerned, between the fourth and thirteenth centuries, there are no archeological or documentary sources attesting to the inhabitation or exploitation of the mines. It was only in the thirteenth century, with the establishment of the Hungarian administration in Transylvania, that the mining areas came into the possession of the royalty, the nobles, or the Catholic Church [6].

In the eighteenth century, the passing of Transylvania under the Austrian administration brought measures aimed at stimulating mining, including the diversification of the types of mined minerals (led, zinc, copper), the construction of accumulation lakes, and the establishment of new mines with paid labor. In Roșia Montană, miners and auxiliary personnel from the golden regions of the Empire were brought without their communities being segregated into separate neighborhoods. However, each group preserved its identity through places of worship, customs, elements of architecture, a popular harbor, and specific furniture [8].

Băița is a village with a rich history that dates back to the Roman period. During the Roman occupation of Dacia (106-272 AD), mining activity was effectively managed and organized throughout the occupied territory. In a short period of time, the richest deposits of native gold (such as those at Barza, Băița, Caraci, Zlatna, Roșia Montană, and Abrud) were identified and partially exploited [9].

The first mining activities were carried out by local peasants, who practiced mining long before the establishment of organized forms of exploitation. Historical documents testify that, in addition to them, foreign settlers and experienced miners, especially from Germany, were brought into the area to participate in the exploitation of the basement resources [10].

It was officially attested in 1270, during the period of early feudalism in Transylvania, when the activity of metal extraction was reactivated in the old mining centers. In the year 1270, miners were brought from the regions that are now part of Germany, France, Austria, and Slovakia and were established in Băița and Vașcău, awaiting the start of gold, silver, and clay mining (at Băița) and the mining of iron ore (at Vașcău) [11].

In the 17th century, Bihor County suffered economic stagnation, becoming under Turkish occupation, and the mines in Băița and Vașcău were occupied. In 1692, with the exit of Oradea from Turkish domination, the Court of Vienna intervened to develop mining in Băița, and in 1726, mining was resumed under the monopoly of the state [12].

In 1951, deposits of rare minerals were identified in the Bihor Mountains, following prospecting by the USSR Ministry of Geology. In the following year, mining began and in addition to Soviet workers and specialists, at that time, approximately 17,000 Romanians were employed in mining [13]. In 1952, the town of Nucet was built from scratch, next to the Băița mine. With the incorporation of communal territories and resources, the new city also assumed responsibility for its administration, including the governance of neighboring villages [13].

In Romania, a limited number of studies have been conducted investigating genetic diversity through the analysis of the human Y chromosome, and in these studies, the number of markers analyzed has been restricted. The purpose of this study is to analyze the genetic profiles of two populations in the Apuseni Mountains area, Băița and Roșia Montană. The main objective is to investigate genetic diversity by analyzing allele frequencies, haplotype frequencies, calculating forensic parameters based on 27 Y-STR loci, and presenting the main haplogroups identified based on Y-STR markers.

## Materials and methods

Data collection

**Figure 1:**
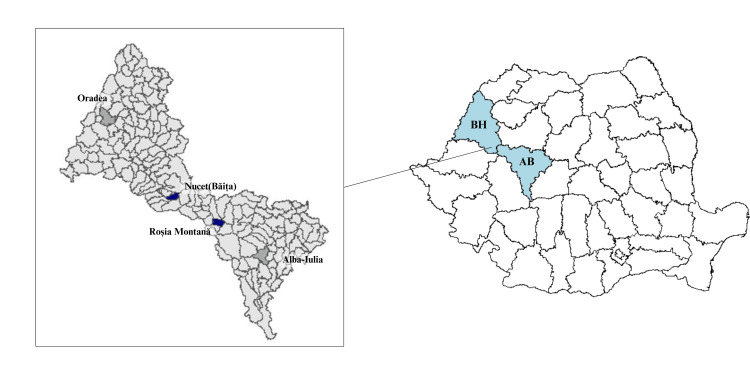
The geographical representation of the towns of Roșia Montană (district of Alba) and Băița, Nucet (district of Bihor). Map created in R, the package used ggplot2, shapefiles provided by DIVA-GIS (https://www.diva-gis.org, Accessed April 26, 2024).

The samples were collected using buccal swabs from 60 unrelated males who have lived in the Apuseni Mountains area (Figure 1) for at least two generations. Men from Roșia Montană area (n = 30) and Băița (n = 30) participated in this study voluntarily, giving their informed written consent to participation. The study was approved by the Ethics Committee of the Faculty of Medicine and Pharmacy at the University of Oradea, Romania.

Laboratory analysis

Extraction and Quantification

For the purpose of genetic analysis, the genetic material was extracted using the MasterPure™ Complete DNA and RNA Purification Kit, EPICENTRE®, using the Cell Samples-Buccal Cells method. The extracted DNA was quantified and evaluated from a purity point of view by the spectral photometric method using the NanoPhotometer™ Pearl from Implen by measuring the absorption at 260 nm, 280 nm, and 230 nm, according to the manufacturer’s working instructions.

Amplification, Capillary Electrophoresis, and Data Interpretation

Amplification was performed on 25 STR markers of the Y chromosome (DYS576, DYS389I, DYS635, DYS389II, DYS627, DYS460, DYS458, DYS19, Y GATA H4, DYS448, DYS391, DYS456, DYS390, DYS438, DY392, DYS518, DYS570, DYS437, DY385, DY449, DYS393, DY439, DY481, DYF387S1, and DYS8533) using the AmpF1STR® Yfiler™ Plus PCR Reagents kit, Applied Biosystems™. The amplified products were migrated through capillary electrophoresis into the 3500 HID Genetic Analyzer, Applied Biosystems™, using consumables and reagents according to the manufacturer’s working instructions. The fluorescence data was taken from 3500 Data Collection Software, Applied Biosystems™. The data were exported and interpreted using GeneMapper® ID-X, Applied Biosystems™ software.

Statistical analysis

Population Genetic Parameters and Forensic Parameters

The allele frequency was determined by direct calculation, representing the number of alleles in relation to the population size of the sample [14]. The frequency of the haplotype was determined by direct counting.

The diversity of the genes was calculated using the formula of Nei [15],$GD=\frac{n}{n-1}\cdot (1-\sum {p_{i}}^{2})$, where $p_{i}$ is the frequency of the allele *i*, and haplotype diversity (HD) was computed using the form $HD=\frac{n}{n-1}\cdot (1-\sum {p_{i}}^{2})$, where $p_{i}$ represents the calculated frequencies of the haplotype *i,* and *n* represents the number of samples analyzed [16].

Forensic parameters such as gene diversity (GD), polymorphism information content (PIC), match probability (MP), and power of discrimination (PD) were calculated using the online program STRAF 2.1.5 [17].

The haplotype match probability (HMP) was estimated as HMP=∑p2i𝐻𝑀𝑃=∑𝑝𝑖2, where 𝑝𝑖 is the frequency of i haplotype [18]. Discrimination capacity (DC) was calculated by dividing the number of different haplotypes by the total sum of the identified haplotypes [19].

HD, HMP, and DC were evaluated in four different levels, including 9 Y-STR loci for the minimal haplotype (Minimal) (which comprise DYS19, DYS389I, DYS389II, DY390, DYS391, DYS392, DYS393, DYS385) for Y12 (Minimal + DYS437, DYS438, DYS439), for Y17-Applied Biosystems Yfiler (Y12+ DYS448, DYS456, DYS458, DY635, YGATAH4) and Y27-Appended Biosystem (Applied Biosystems Yfiler + DY576, DY627, DY460, DY518, DYS570, DY449, DYS481, DYF387S1, DYS533) [20].

Haplogroup Prediction

The predictions of haplogroups from the Y-STR values of the two batches were made using Athey’s haplogroup predictor [21]. In this study, the markers DYS627, DYS518, and DY387S1 were excluded from the analysis due to the lack of allele frequency data for these markers. The batch program used 111 markers from the American-based Family Tree DNA (FTDNA) set to perform the prediction, with Eastern Europe selected as the area.

## Results

Allele frequency and forensic parameters for 27 de loci Y-STR

**Figure 2:**
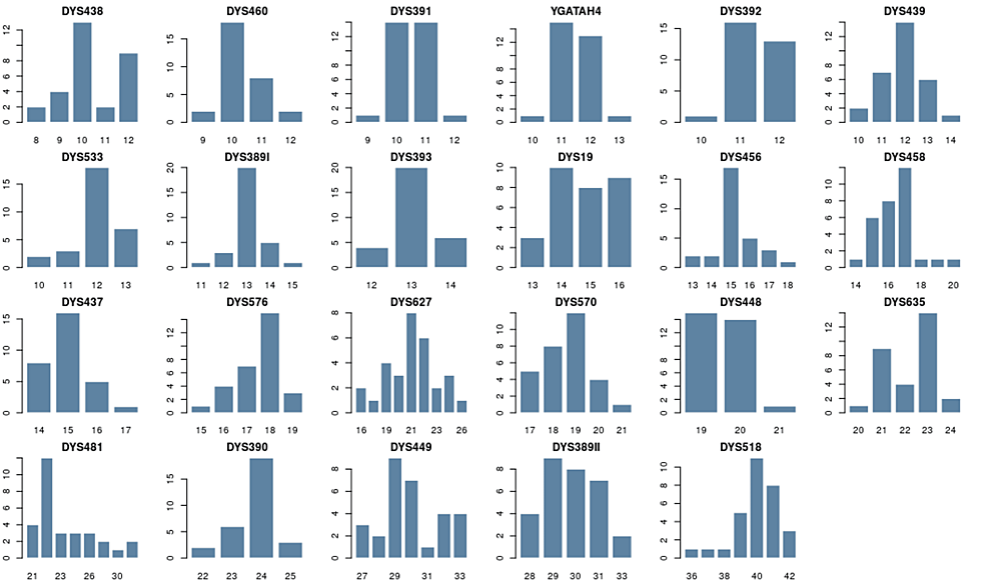
Allele frequency distribution for 23 Y-STR locations in the population of Băița. The horizontal scale indicates the allelic values of each locus, and the vertical scale represents the frequency of occurrence of each allele. Figure created with STRAF. STARF: STR Analysis for Forensics

Through the analysis of the Y-STR profiles, it was determined that there were 117 single-copy alleles for the batch from Băița and 108 single-copy alleles for the batch from Roșia Montană. The frequency of alleles in the batch at Băița is between 0.033 and 0.667, the least allelic variants (n = 3) were observed in the DYS448, DYS392, and DYS393 loci, while the most allelic variants were recorded for the DYS627 loci (n = 9). The allele frequency for 27 Y-STR loci is presented in Appendices, Table 4 and the allele frequency distribution for 23 Y-STR is represented in Figure 2.

**Figure 3:**
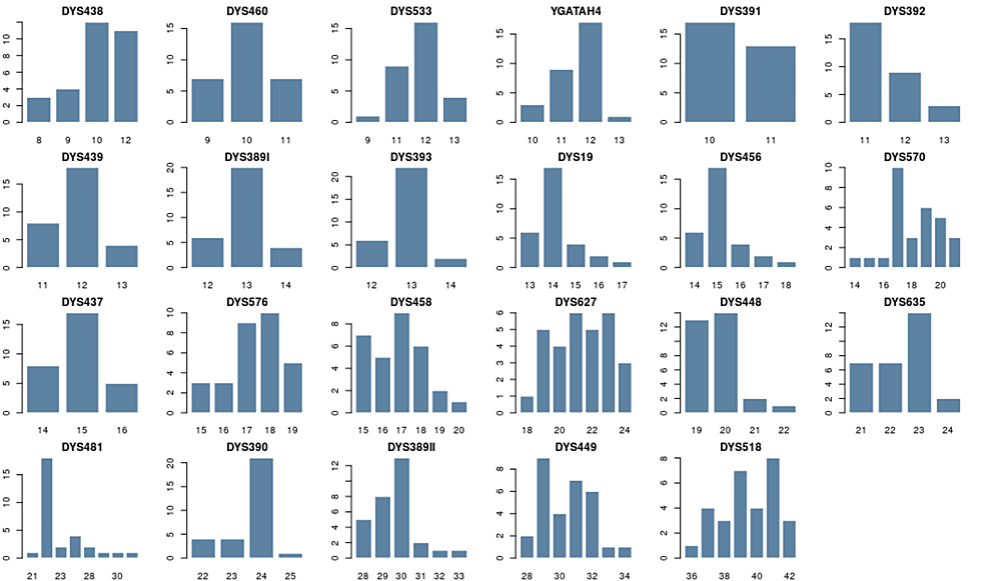
Allele frequency distribution for 23 Y-STR loci in the population of Roșia Montană. The horizontal scale indicates the allelic values of each locus, and the vertical scale represents the frequency of occurrence of each allele. Figure created with STRAF. STARF: STR Analysis for Forensics

In the Roșia Montană batch, the allele frequency ranges from 0.033 to 0.733, and the fewest allele variants (n = 2) were observed at the DYS391 locus, whereas the most variations (n = 8) were seen at DYS570 and DYS481. The allele frequency for 27 Y-STR loci is presented in Supplementary Material Table 7. The allele frequency distribution for 23 Y-STR is represented in Figure 3 for the population from Roșia Montană.

In the case of the multi-locus marker DYS385a/b, 17 haplotype combinations with 11 different alleles were identified in the batch at Băița, and 17 haplotype combinations with nine different alleles each were identified in the batch at Roșia Montană. At the same time, the DYF387S1 locus presented 14 haplotypes formed from combinations of six distinct alleles in the batch at Băița and 12 haplotypes formed from a combination of eight distinct alloys in the batch at Roșia Montană. Also, in the lot from Roșia Montană was identified an individual that presented three variants on locus DYF387S1.

In both datasets, all variant alleles were within the allelic scale range specific to the AmpF1STR® Yfiler PlusTM PCR Reagents™ kit amplification kit, Applied Biosystems™.

GD, PIC, MP, and PD are presented in Supplementary Material, Tables 4 and 7. The GD in the batch from Băița ranges from 0.515 for DYS393 to 0.947 for DYS385. The highest values of GD were recorded at rapidly mutating (RM ) markers, reaching 0.917 at DYF387S1, 0.869 at DYS627, and 0.832 at DYS449. Also, the highest GD values for single-copy Y-STR markers were 0.809 for DYS481 and 0.789 for DYS389II. By contrast, the lowest GD values were 0.533 for the DYS389I and 0.545 for the DYS392. GD ranges from 0.432 for DYS393 to 0.931 for the DYS385 locus in the Roșia Montană lot. Also, in this case, the highest values for GD were recorded at RM markers, respectively: 0.864 at DYS627, 0.848 at DYF387S1, and 0.846 at DYS518. As for single copy markers, the highest value of GD was 0.809 for DYS458, and the lowest values were 0.490 for DYS390 and 0.508 for DYS391.

The PIC values varied between 0.419 for the DYS392 and 0.910 for the DYS385 in the population of Băița and between 0.370 for the DYS393 and 0.893 for the DYS385 in the Roșia Montană population.

In the population of Băița, the lowest MP values were recorded for the multi-copy marker, DYS385, with a value of 0.084, and for the RM markers, DYF387S1, with a value of 0.113, and DYS627, with a value of 0.160. At the same time, the highest values were recorded with the markers DYS389I, with a value of 0.484, and DYS393, with a number of 0.502. In the batch from Roșia Montană, the lowest MP values were 0.100 for the multi-copy locus, DYS385, and 0.164 for the RM locus, DYS627, respectively, and 0.180 for the RM location, DYF387S1. At the same time, the highest values were recorded for single-copy markers, reaching 0.527 for the DYS390 and 0.582 for the DYS393.

The PD values vary between 0.498 and 0.916 in the batch at Băița, respectively, and between 0.418 and 0.900 in the batch at Roșia Montană, for the same loci, DYS393 and DYS385.

Haplotype frequency

The genetic diversity of the population examined, consisting of 60 individuals from Roșia Montană and Băița, as well as their genotypic profiles, are summarized in Supplementary Tables 3 and 6. In this study, the genetic diversity of two distinct populations was evaluated using genetic analyses based on different markers. First, analysis was carried out using 27 Y-STR markers, and it was observed that all individuals in Băița presented unique genotypic profiles, resulting in an HMP of 0.033 and an HD of 1.000. In contrast, in the population of Roșia Montană, four identical haplotypes were identified in two individuals each, resulting in an HMP of 0.042 and an HD of 0.991.

Subsequently, the number of markers was reduced to 17, and it was found that the results for the population of Băița remained constant. However, using the minimal marker kit, a decrease in the number of unique haplotypes was observed, indicating a discriminatory capacity (DC) of 0.900. In the case of the population of Roșia Montană, analysis with the minimal kit, Y12 and Y17, resulted in a significant decrease in the number of unique haplotypes, with HD ranging between 0.959 and 0.982, while DC decreased from 0.867 to 0.733.

Table 1 provides an overview of the genetic parameters evaluated in the Băița population using four distinct levels of Y-STR markers.

**Table 1:** Genetic parameters evaluated in the population of Băița for the minimal set Y12, Y17, and Y27.

	Minimal	Y12	Y17	Y27
Haplotype match probability (HMP)	0.040	0.038	0.033	0.033
Haplotype diversity (HD)	0.993	0.995	1.000	1.000
Total haplotypes	30	30	30	30
Unique haplotypes	24	26	30	30
Identical haplotype in two individuals	3	2	-	-
Discrimination capacity (DC)	0.900	0.933	1.000	1.000

Table 2 presents the same analysis for the Roșia Montană population, using four distinct levels of Y-STR markers.

**Table 2:** Genetic parameters evaluated in the population of Roșia Montană for the Minimal set Y12, Y17, and Y27.

	Minimal	Y12	Y17	Y27
Haplotype match probability (HMP)	0.073	0.071	0.051	0.042
Haplotype diversity (HD)	0.959	0.961	0.982	0.991
Total haplotypes	30	30	30	30
Unique haplotypes	18	20	20	22
Identical haplotype in two individuals	3	2	2	4
Identical haplotype in three individuals	-	-	2	-
Identical haplotype in six individuals	1	1	-	-
Discrimination capacity (DC)	0.733	0.767	0.800	0.867

Haplogroup frequency

The haplogroup prediction was made using the Whit Athey Predictor for 23 Y-STR markers. The accuracy of the haplogroup predictions was 100%, except in two cases where the precision was 99.9% (Roșia Montană) and 93.5% (Băița). The predicted haplogroups for the population of Băița and Roșia Montană using haplogroup predictor are shown in Supplementary Material Table 5 and Table 8, respectively.

**Figure 4:**
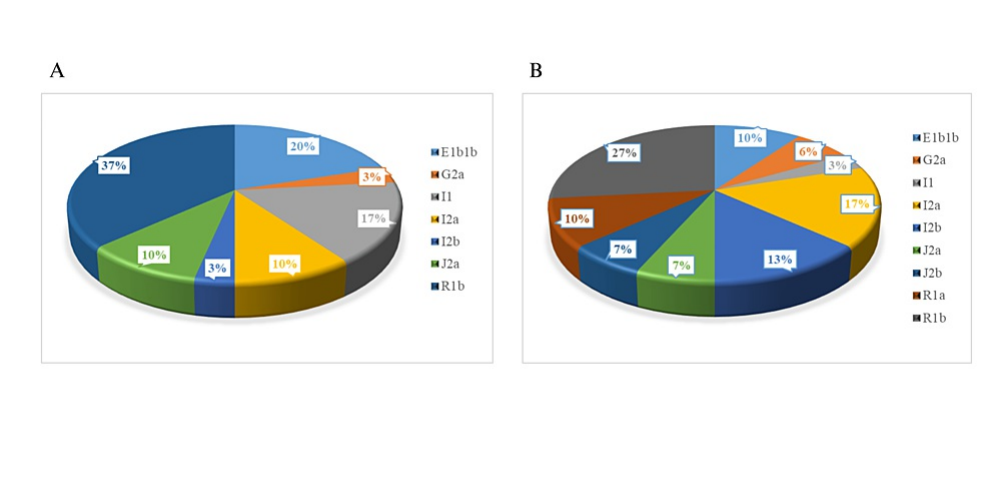
(A) Distribution of haplogroups in the batch Roșia Montană. (B) Distribution of haplogroups in batch Băița. The image was created in Microsoft Excel by the authors of this article and represents the distribution of haplogroups both in Roșia Montană and in Băița.

A total of nine haplogroups was identified in the batch at Băița, while only seven were observed in the batch at Roșia Montană. This indicates a greater diversity of haplogroups in the batch at Băița than in that at Roșia Montană, despite the fact that both batches are based on the same five major haplogroups (E, G, I, J, and R). The distribution of haplogroups in the two batches is shown in Figure 4.

In the Roșia Montană community, the most common haplogroup is R1b, with a total of 11 individuals, followed by E1b1b with six individuals. On the other hand, G2a and J2b are completely absent, while J2a and I2b are rare, with only three and one individual, respectively.

In contrast, in the Băița community, haplogroup R1b remains predominant, with eight individuals, but with a lower frequency than in Roșia Montană. Interestingly, G2a, with two individuals, and I2b, with four individuals, are more present in Băița than in Roșia Montană. Also, J2b and R1a are present only in Băița and absent in Roșia Montană.

Haplogroups I1 and I2a have a different distribution between the two communities. In Roșia Montană, I1 has a significant frequency, with five individuals, while in Băița it is much rarer, with one individual. Instead, I2a has a greater presence in Băița, with five individuals, compared to the three in Roșia Montană.

## Discussion

In this study, we aimed to present the genetic diversity through the analysis of the Y chromosome of two populations in the Apuseni Mountains, inhabitants of the Băița area and inhabitants of the Roșia Montană area.

Genetic diversity within a population is often assessed by measuring expected heterozigotism, which represents the probability that two samples taken from the same population at a particular location of the genome will have different genetic types. Expected heterozygosity is a sensitive measure of genetic diversity, with higher values indicating greater diversity [22]. The GD in Băița and Roșia Montană is evident, with the markers with rapid mutation showing the highest values: DYF387S1, DYS627, and DYS518 in Roșia Montană, respectively DYF387S1, DYS627, and DYS449 in Băița. In both populations, single-copy markers such as DYS481 and DYS458 showed significant levels of diversity. However, there are also markers with lower diversity, such as DYS389I and DYS392 in Băița, and DYS390 and DYS391 in Roșia Montană.

The PIC is assessed according to the genetic marker’s ability to identify variability in the population, based on the number and frequency of alleles detected. Therefore, the PIC reflects the marker’s DC, influenced by the number and distribution of the frequency of the identified alleles [23], [24]. Marker DYS385 was observed to have the highest PIC values in both populations, suggestive of significant genetic diversity at this locus, while marker DYS392 had lower PIC values, indicating less genetic diversity at this locus.

MP is the probability of obtaining a match between two distinct and unrelated individuals and provides a useful measure for assessing the discriminatory power of the DNA profiling system [25]. The low MP values, especially at markers DYS385 and RM DYF387S1 and DYS627 in the batch from Băița and Roșia Montană, indicate a good ability to distinguish between individuals at these loci. Conversely, higher MP values at markers DYS389I, DYS390, and DYS393 show a higher probability of coincidence between individuals in both populations.

The PD is a measure used in genetics to determine the ability of a set of genetic markers to differentiate between individuals. It quantifies the probability that two randomly chosen individuals have different haplotypes (combinations of alleles at multiple genetic locations) [26]. PD was observed to show minimum values at marker DYS393 and maximum values at marker DYS385 in both populations.

In the analysis of HD, similar values of HD for the minimal set of markers were also reported in the study [27], which analyzed genetic diversity in Romania. In this study, 97 different haplotypes were identified, of which 92 were unique. The HD was 0.9887, and the DC was 0.9326. These results suggest that Băița and the general population in Romania show similar levels of genetic diversity in terms of the minimum set of markers used in the study. In contrast, the diversity of 0.959 for Roșia Montană, although high, is still smaller than that in Băița. This may suggest a less genetically varied population.

During the set of 12 markers in the population of Roșia Montană, 20 unique haplotypes were identified out of a total of 30 samples, while in Băița, 26 unique haplotypes were also found out of 30 samples. These results suggest substantial genetic diversity in both populations. A similar study conducted in Transylvania [28] compared two populations and analyzed 175 samples, observing 134 different haplotypes. Of the 86 samples in Miercurea Ciuc, 81 were unique haplotypes, and for Lunca de Sus, out of 54 samples, 39 were unique.

By extending the analysis to 17 locations, it was found that the number of unique haplotypes remained constant in Roșia Montană, while in Băița, this number increased to 30. This result suggests a potentially greater genetic diversity in Băița compared to Roșia Montană, while the genetic structure of this community remained stable. A similar study conducted in Bucharest [29] analyzed a batch of 122 randomly chosen men from nine counties in southern Romania. A total of 115 different haplotypes were determined, of which 109 were unique. A similar result of genetic diversity was observed in another study conducted in eastern Romania [30], which found a diversity of haplotypes of 0.9895 for the batch of 54 males from Romania. Also, another study conducted in several areas of Romania (Cluj, Brașov, Dolj, Mehedinți) [31] showed similar values for a larger number of markers (19 markers), with diversities of haplotypes ranging between 0.9775 and 0.9973.

In the analysis of 27 Y-STR markers, the highest values of HD and capacity for discrimination were recorded. For Roșia Montană, HD was 0.991 and DC was 0.867, while for Băița, both values were 1.000. Also, in another study that analyzed 23 Y-STR markers [32], an HD of 0.9995 was obtained. Thus, the use of an extended set of markers can bring greater precision and accuracy to the genetic analysis of populations.

In both populations from Băița and Roșia Montană, the same five major haplogroups (E, G, I, J, and R) were identified. In Roșia Montană, R1b is the most common haplogroup, followed by E1b1b. G2a and J2b are completely absent, and J2a and I2b are rare. In Băița, R1b remains predominant, but with a lower frequency than in Roșia Montană. G2a and I2b are more present in Băița, and J2b and R1a are present only there. The distribution of haplogroups I1 and I2a is different between the two communities, with I1 more frequent in Roșia Montană and I2a more present in Băița.

The haplogroup R of the Y chromosome is one of the 20 haplogroups that make up the standardized global phylogenesis. It consists of two main components: R1-M173 and R2-M479. Within R1-M173, most of the variation existing in Eurasia is restricted to R1a-M420 and R1b-M343. In Europe, haplogroup R1a is most commonly found in Eastern Europe, while R1b predominates in Western Europe [33].

In Romania, haplogroup R1a has been observed to be common in several regions, including in the population of Șinteu, north-western Romania [34], the clusters of Cluj, Mehedinți, Dolj, and Brașov, but also in the cluster of Romanians in eastern Romania [30]. In contrast, haplogroup R1b was to be dominant in the population of Palota, Bihor [34], as well as in Piatra Neamț and Buhuși [30].

Haplogroup E1b1 is now subdivided into two distinct basal branches: E-V38 (E1b 1a) and E-M215 (E 1b 1b), together with E-M329 (former E1b1c). Each of these two subdivisions has a specific geographical distribution. The haplogroup E-M2 is most common in sub-Saharan Africa, while the haplogroup E-M329 has been identified mainly in eastern Africa. The other basal branch of haplogroup E1b1, E-M215, is spread geographically from southern Europe to northern and eastern Africa and is suggested to have originated in this region [35]. In the Roșia Montană batch, haplogroup E1b1 was identified in 20% of the males. The same haplogroup was also observed in lots in Cluj, Dolj, and Brașov [31].

Haplogroup I-M170 is part of the European genetic heritage of Y chromosomes, accounting for about 18% of the total paternal lines on average. Its virtual absence in other regions, including the Middle East, suggests that it appeared in Europe, probably before the Last Glacial Maxim. Haplogroup I1a is predominant in northern Europe, with the highest frequency in the Scandinavian populations, while haplogroup I1b* is the most common clade in Eastern Europe and the Balkans [36]. Haplogroup I2a, currently predominant in Eastern Europe, has been identified in ancient Y-STR sequences from hunter-gatherers from Switzerland, Hungary, and Scandinavia, as well as in samples from the Neolithic and Bronze Age in Hungary and Germany [37]. The third most common haplogroup found in the population of Roșia Montană is I1, which is also in Harghita County, Transylvania [32]. On the other hand, haplogroups I2a and I2b were commonly found in the population of Băița. These haplogroups have also been identified in the populations of Cluj, Mehedinți, Dolj, Brașov [31], Odorheiu Secuiesc [32], Piatra-Neamț, and Buhuși [30].

Haplogroup J consists of two main clads: J-M267(J1) and J-M172(J2). J-M172, although as common as J-M267 in some populations in the Middle East, is more widespread in Europe [38]. In the two populations studied, the frequency of haplogroup J2a is 10% in Roșia Montană and 7% for both J2a and J2b in the population of Băița. In addition, in Romania, the frequencies of haplogroup J2a were in Odorheiu Secuiesc [32] and J2b in Mehedinți [31].

## Conclusions

In this study, we investigated the genetic diversity by analyzing the Y chromosome, using 27 Y-STR markers, in two populations from the Apuseni Mountains: the inhabitants of the Băița area and the inhabitants of the Roșia Montană area. These two towns are remarkable for their fascinating history. Analysis of Y-STR profiles revealed significant genetic diversity in both studied groups. The maximum values of GD are recorded at the RM markers in both populations. In this study, the genetic diversity of two distinct populations was assessed using genetic analyses based on different markers from the minimal kit to Y27. The use of a larger number of Y-STR markers has been observed to demonstrate greater discriminatory power and HD than kits with a lower number of markers. In terms of haplogroup distribution, the most common determining haplogroups in the population of Roșia Montană are R1b, E1b1b, and I1, while in Băița, the haplogroups R1b, I2a, and I2b prevail. This study can be useful by providing a deeper understanding of the genetic diversity and genetic structure of the population of Roșia Montană and Băița. This could serve as a basis for future research in areas such as population genetics and genealogy.

## Appendices

**Table 3:** Haplotypes for 27 Y chromosomal STR loci in the population of Băița. STR: short tandem repetitions

ID	Population name	DYS576	DYS389I	DYS635	DYS389II	DYS627	DYS460	DYS458	DYS19	YGATAH4	DYS448	DYS391	DYS456	DYS390	DYS438	DYS392	DYS518	DYS570	DYS437	DYS385	DYS449	DYS393	DYS439	DYS481	DYF387S1	DYS533
BA_103	Băița	18	13	24	30	16	11	15	15	12	19	10	16	25	11	11	39	18	14	11, 14	32	13	11	23	36, 39	12
BA_104	Băița	16	12	20	28	19	10	15	14	11	20	11	13	23	10	11	40	20	16	14, 14	27	14	11	25	37, 38	11
BA_105	Băița	18	13	23	29	24	10	17	14	12	19	11	15	24	12	12	41	17	15	11, 13	30	13	13	22	35, 36	12
BA_106	Băița	18	14	23	30	21	10	17	14	12	19	11	15	24	12	12	40	18	15	11, 13	29	13	12	21	35, 36	12
BA_107	Băița	18	13	23	30	22	10	17	14	11	19	10	15	24	12	12	42	17	15	11, 13	29	13	12	22	35, 36	12
BA_108	Băița	17	14	22	31	21	10	16	13	12	20	10	16	24	10	11	41	19	14	16, 18	33	13	12	22	35, 38	13
BA_109	Băița	15	12	21	28	24	10	16	16	10	20	10	15	22	10	10	41	18	17	15, 19	29	14	12	21	37, 38	10
BA_110	Băița	17	13	23	31	21	10	18	15	11	19	11	15	24	10	11	40	19	15	14, 14	33	13	14	31	37, 39	13
BA_111	Băița	18	13	23	29	24	10	17	14	12	19	11	15	24	12	12	42	19	15	12, 14	28	13	13	22	35, 36	12
BA_112	Băița	16	12	21	28	19	10	16	15	11	19	10	13	24	9	11	39	18	16	10, 18	30	12	12	22	37, 38	11
BA_113	Băița	18	13	23	33	20	10	17	16	11	20	11	15	24	10	11	40	17	15	14, 16	33	13	13	29	39, 39	13
BA_114	Băița	19	13	21	29	21	11	16	15	11	20	10	14	23	10	12	38	19	14	15, 16	27	13	12	25	36, 38	13
BA_115	Băița	18	13	24	31	20	10	17	16	11	19	10	15	23	10	11	39	19	15	14, 15	30	13	12	30	38, 39	12
BA_116	Băița	18	13	23	28	26	10	17	14	12	19	11	15	24	12	12	40	19	16	11, 15	29	13	12	22	35, 37	12
BA_117	Băița	18	13	23	29	22	10	17	14	12	19	11	15	24	12	12	41	18	15	11, 13	29	13	12	22	35, 36	12
BA_118	Băița	19	13	21	29	21	11	19	15	12	21	11	15	22	10	11	36	19	16	13, 14	30	14	13	21	40, 40	10
BA_119	Băița	18	13	21	29	20	11	16	14	11	19	10	15	23	9	11	39	19	15	10, 16	29	12	11	22	37, 38	11
BA_120	Băița	18	13	23	29	23	10	17	14	12	19	10	15	24	12	12	41	18	15	11, 14	29	13	11	22	35, 37	12
BA_121	Băița	17	14	22	30	22	11	15	16	11	20	10	17	24	8	12	41	20	15	15, 15	30	14	11	26	39, 40	12
BA_122	Băița	18	13	21	29	22	12	15	16	11	20	11	17	23	10	12	40	19	15	15, 15	27	14	11	26	36, 37	13
BA_123	Băița	17	13	22	29	21	10	14	15	11	20	9	16	23	9	11	37	18	14	13, 16	30	12	13	22	38, 38	12
BA_124	Băița	19	13	23	30	22	9	16	13	12	20	10	17	24	10	11	40	21	14	16, 18	32	13	12	23	35, 39	12
BA_125	Băița	18	15	23	31	23	10	17	14	12	19	11	15	24	12	12	40	18	15	11, 13	29	13	12	22	35, 36	12
BA_126	Băița	17	13	23	31	17	11	17	16	12	20	10	16	25	12	11	42	17	14	9, 14	33	13	10	25	36, 38	12
BA_127	Băița	18	13	23	31	19	10	16	16	11	19	11	15	24	10	11	39	19	15	14, 15	30	13	12	31	38, 39	13
BA_128	Băița	16	14	23	31	16	11	15	15	12	20	11	15	25	11	12	41	20	14	11, 14	32	13	10	23	37, 38	12
BA_129	Băița	17	14	22	30	21	12	16	16	11	20	10	18	24	8	12	40	20	15	15, 15	29	14	11	26	39, 40	12
BA_130	Băița	17	13	21	30	22	9	20	13	13	20	10	16	24	10	11	41	19	14	16, 18	31	13	12	22	36, 38	13
BA_131	Băița	18	13	21	30	19	10	17	16	11	20	11	15	24	10	11	40	19	15	14, 15	32	13	13	29	38, 39	12
BA_132	Băița	16	11	21	33	21	11	15	15	11	19	12	14	24	9	11	40	17	16	14, 17	28	12	12	21	36, 38	12

**Table 4:** Allele frequencies and forensic parameters for 27 Y-STR loci in the Băița population. N: number of samples; Nall: number of alleles; GD: gene diversity; PIC: polymorphism information content; MP: match probability; PD: power of discrimination; STRs: short tandem repetitions

Allele/n	DYS576	DYS389I	DYS635	DYS389II	DYS627	DYS460	DYS458	DYS19	YGATAH4	DYS448	DYS391	DYS456	DYS390	DYS438	DYS392	DYS518	DYS570	DYS437	DYS449	DYS393	DYS439	DYS481	DYS533		Allele/n	DYS385		Allele/n	DYF387S1
8														0.067											9, 14	0.033		35, 36	0.200
9						0.067					0.033			0.133											10, 16	0.033		35, 37	0.067
10						0.600			0.033		0.467			0.433	0.033						0.067		0.067		10, 18	0.033		35, 38	0.033
11		0.033				0.267			0.500		0.467			0.067	0.533						0.233		0.100		11, 13	0.167		35, 39	0.033
12		0.100				0.067			0.433		0.033			0.300	0.433					0.133	0.467		0.600		11, 14	0.100		36, 37	0.033
13		0.667						0.100	0.033			0.067								0.667	0.200		0.233		11, 15	0.033		36, 38	0.133
14		0.167					0.033	0.333				0.067						0.267		0.200	0.033				12, 14	0.033		36, 39	0.033
15	0.033	0.033					0.200	0.267				0.567						0.533							13, 14	0.033		37, 38	0.167
16	0.133				0.067		0.267	0.300				0.167						0.167							13, 16	0.033		37, 39	0.033
17	0.233				0.033		0.400					0.100					0.167	0.033							14, 14	0.067		38, 38	0.033
18	0.500						0.033					0.033					0.267								14, 15	0.100		38, 39	0.100
19	0.100				0.133		0.033			0.500							0.400								14, 16	0.033		39, 39	0.033
20			0.033		0.100		0.033			0.467							0.133								14, 17	0.033		39, 40	0.067
21			0.300		0.267					0.033							0.033					0.133			15, 15	0.100		40, 40	0.033
22			0.133		0.200								0.067									0.400			15, 16	0.033			
23			0.467		0.067								0.200									0.100			15, 19	0.033			
24			0.067		0.100								0.633												16, 18	0.100			
25													0.100									0.100							
26					0.033																	0.100							
27																			0.100										
28				0.133															0.067										
29				0.300															0.300			0.067							
30				0.267															0.233			0.033							
31				0.233															0.033			0.067							
32																			0.133										
33				0.067															0.133										
36																0.033													
37																0.033													
38																0.033													
39																0.167													
40																0.367													
41																0.267													
42																0.100													
N	30	30	30	30	30	30	30	30	30	30	30	30	30	30	30	30	30	30	30	30	30	30	30		N	30		N	30
Nall	5	5	5	5	9	4	7	4	4	3	4	6	4	5	3	7	5	4	7	3	5	8	4		Nall	17		Nall	14
GD	0.690	0.533	0.692	0.789	0.869	0.579	0.749	0.743	0.579	0.549	0.582	0.653	0.563	0.720	0.545	0.779	0.747	0.637	0.832	0.515	0.706	0.809	0.591		GD	0.947		GD	0.917
PIC	0.621	0.479	0.615	0.722	0.822	0.501	0.681	0.664	0.464	0.421	0.465	0.599	0.500	0.647	0.419	0.716	0.677	0.554	0.778	0.445	0.633	0.761	0.520		PIC	0.910		PIC	0.877
MP	0.333	0.484	0.331	0.238	0.160	0.440	0.276	0.282	0.440	0.469	0.438	0.369	0.456	0.304	0.473	0.247	0.278	0.384	0.196	0.502	0.318	0.218	0.429		MP	0.084		MP	0.113
PD	0.667	0.516	0.669	0.762	0.840	0.560	0.724	0.718	0.560	0.531	0.562	0.631	0.544	0.696	0.527	0.753	0.722	0.616	0.804	0.498	0.682	0.782	0.571		PD	0.916		PD	0.887

**Table 5:** Haplogroups predicted for the Băița population using Athey’s haplogroup predictor by 23 Y-STRs. STR: short tandem repetitions

ID	Haplotype	DYS393	DYS390	DYS19	DYS391	DYS385a	DYS385b	DYS439	DYS389I	DYS392	DYS389II	DYS458	DYS437	DYS448	DYS449	DYS460	YGATAH4	DYS456	DYS576	DYS570	DYS438	DYS481	DYS533	DYS635	Haplogroup	Fitness score	Probability (%)
BA_103	103	13	25	15	10	11	14	11	13	11	30	15	14	19	32	11	12	16	18	18	11	23	12	24	R1a	44	100
BA_104	104	14	23	14	11	14	0	11	12	11	28	15	16	20	27	10	11	13	16	20	10	25	11	20	I1	27	100
BA_105	105	13	24	14	11	11	13	13	13	12	29	17	15	19	30	10	12	15	18	17	12	22	12	23	R1b	34	100
BA_106	106	13	24	14	11	11	13	12	14	12	30	17	15	19	29	10	12	15	18	18	12	21	12	23	R1b	30	100
BA_107	107	13	24	14	10	11	13	12	13	12	30	17	15	19	29	10	11	15	18	17	12	22	12	23	R1b	39	100
BA_108	108	13	24	13	10	16	18	12	14	11	31	16	14	20	33	10	12	16	17	19	10	22	13	22	E1b1b	43	100
BA_109	109	14	22	16	10	15	19	12	12	10	28	16	17	20	29	10	10	15	15	18	10	21	10	21	G2a	21	100
BA_110	110	13	24	15	11	14	0	14	13	11	31	18	15	19	33	10	11	15	17	19	10	31	13	23	I2a	34	100
BA_111	111	13	24	14	11	12	14	13	13	12	29	17	15	19	28	10	12	15	18	19	12	22	12	23	R1b	30	100
BA_112	112	12	24	15	10	10	18	12	12	11	28	16	16	19	30	10	11	13	16	18	9	22	11	21	J2b	34	100
BA_113	113	13	24	16	11	14	16	13	13	11	33	17	15	20	33	10	11	15	18	17	10	29	13	23	I2a	43	100
BA_114	114	13	23	15	10	15	16	12	13	12	29	16	14	20	27	11	11	14	19	19	10	25	13	21	I2b	38	100
BA_115	115	13	23	16	10	14	15	12	13	11	31	17	15	19	30	10	11	15	18	19	10	30	12	24	I2a	43	100
BA_116	116	13	24	14	11	11	15	12	13	12	28	17	16	19	29	10	12	15	18	19	12	22	12	23	R1b	31	100
BA_117	117	13	24	14	11	11	13	12	13	12	29	17	15	19	29	10	12	15	18	18	12	22	12	23	R1b	35	100
BA_118	118	14	22	15	11	13	14	13	13	11	29	19	16	21	30	11	12	15	19	19	10	21	10	21	G2a	29	100
BA_119	119	12	23	14	10	10	16	11	13	11	29	16	15	19	29	11	11	15	18	19	9	22	11	21	J2a	30	93.5
BA_120	120	13	24	14	10	11	14	11	13	12	29	17	15	19	29	10	12	15	18	18	12	22	12	23	R1b	35	100
BA_121	121	14	24	16	10	15	0	11	14	12	30	15	15	20	30	11	11	17	17	20	8	26	12	22	I2b	25	100
BA_122	122	14	23	16	11	15	0	11	13	12	29	15	15	20	27	12	11	17	18	19	10	26	13	21	I2b	28	100
BA_123	123	12	23	15	9	13	16	13	13	11	29	14	14	20	30	10	11	16	17	18	9	22	12	22	J2a	32	100
BA_124	124	13	24	13	10	16	18	12	13	11	30	16	14	20	32	9	12	17	19	21	10	23	12	23	E1b1b	43	100
BA_125	125	13	24	14	11	11	13	12	15	12	31	17	15	19	29	10	12	15	18	18	12	22	12	23	R1b	29	100
BA_126	126	13	25	16	10	9	14	10	13	11	31	17	14	20	33	11	12	16	17	17	12	25	12	23	R1a	29	100
BA_127	127	13	24	16	11	14	15	12	13	11	31	16	15	19	30	10	11	15	18	19	10	31	13	23	I2a	42	100
BA_128	128	13	25	15	11	11	14	10	14	12	31	15	14	20	32	11	12	15	16	20	11	23	12	23	R1a	40	100
BA_129	129	14	24	16	10	15	0	11	14	12	30	16	15	20	29	12	11	18	17	20	8	26	12	22	I2b	21	100
BA_130	130	13	24	13	10	16	18	12	13	11	30	20	14	20	31	9	13	16	17	19	10	22	13	21	E1b1b	34	100
BA_131	131	13	24	16	11	14	15	13	13	11	30	17	15	20	32	10	11	15	18	19	10	29	12	21	I2a	48	100
BA_132	132	12	24	15	12	14	17	12	11	11	33	15	16	19	28	11	11	14	16	17	9	21	12	21	J2b	24	100

**Table 6:** Haplotypes for 27 Y chromosomal STR loci in the population of Roșia Montană. STR: short tandem repetitions

ID	Population name	DYS576	DYS389I	DYS635	DYS389II	DYS627	DYS460	DYS458	DYS19	YGATAH4	DYS448	DYS391	DYS456	DYS390	DYS438	DYS392	DYS518	DYS570	DYS437	DYS385	DYS449	DYS393	DYS439	DYS481	DYF387S1	DYS533
RM_002	Roșia Montană	18	13	24	33	19	11	18	17	11	20	11	15	24	10	11	40	18	15	14, 16	32	13	13	29	37, 41	12
RM_003	Roșia Montană	16	12	21	28	19	10	15	14	11	20	10	14	22	10	11	37	21	15	14, 14	28	13	11	28	38, 39	11
RM_004	Roșia Montană	18	13	23	29	23	10	18	14	12	19	11	15	24	12	12	41	17	15	11, 13	29	13	12	22	35, 36	12
RM_005	Roșia Montană	18	13	21	30	20	10	16	14	10	22	10	15	22	9	11	38	19	14	12, 15	29	12	11	22	39, 40	11
RM_006	Roșia Montană	15	12	22	28	19	10	15	15	10	20	10	14	23	8	11	39	20	16	14, 14	31	12	11	26	37, 37	11
RM_007	Roșia Montană	17	14	23	30	21	11	17	14	12	19	11	15	24	12	13	37	17	15	11, 14	31	13	11	22	35, 36	11
RM_035	Roșia Montană	19	13	22	30	21	9	19	13	12	20	10	17	24	10	11	39	20	14	16, 19	31	13	12	23	37, 38	12
RM_043	Roșia Montană	18	13	23	29	23	10	18	14	12	19	11	15	24	12	12	42	17	15	11, 13	29	13	12	22	35, 36	12
RM_044	Roșia Montană	18	13	23	29	24	10	17	14	12	19	11	15	24	12	12	41	17	15	11, 13	29	13	12	22	35, 36	12
RM_045	Roșia Montană	17	14	23	30	21	11	17	14	12	19	11	15	24	12	13	37	17	15	11, 14	31	13	11	22	35, 36	11
RM_067	Roșia Montană	18	13	23	29	24	10	18	14	12	19	11	16	24	12	12	39	19	15	13, 13	29	13	12	22	35, 36	12
RM_070	Roșia Montană	15	12	22	28	19	10	15	15	10	20	10	14	23	8	11	39	20	16	14, 14	31	12	11	26	37, 37	11
RM_074	Roșia Montană	16	13	23	30	21	10	18	13	12	21	11	14	23	9	11	39	17	15	14, 18	31	12	12	22	39, 40	11
RM_076	Roșia Montană	18	13	23	29	23	10	18	14	12	19	11	15	24	12	12	42	17	15	11, 13	29	13	12	22	35, 36	12
RM_077	Roșia Montană	17	13	23	30	20	10	17	16	11	20	10	15	24	10	11	41	16	15	15, 14	32	13	12	30	39, 39	13
RM_079	Roșia Montană	17	13	22	30	21	9	15	13	12	20	10	16	24	10	11	40	19	14	15, 18	34	13	12	22	37, 38	12
RM_081	Roșia Montană	18	13	23	29	24	10	17	14	12	19	11	15	24	12	12	41	17	15	11, 13	29	13	12	22	35, 36	12
RM_086	Roșia Montană	18	13	23	30	22	9	15	14	12	21	10	16	24	10	11	41	21	14	16, 19	32	13	13	23	35, 36, 37	12
RM_087	Roșia Montană	17	13	23	31	22	9	16	13	12	20	10	15	24	10	11	38	20	14	18, 19	32	13	12	22	34, 34	12
RM_089	Roșia Montană	17	12	21	28	18	10	15	14	11	20	10	14	24	10	11	41	20	16	13, 15	31	13	12	28	37, 39	11
RM_091	Roșia Montană	19	13	21	30	23	9	17	14	11	19	10	15	23	9	11	38	15	14	14, 16	33	12	11	21	36, 38	12
RM_092	Roșia Montană	18	13	23	29	23	11	17	14	12	19	11	15	24	12	12	41	17	15	11, 13	29	13	12	22	35, 36	12
RM_093	Roșia Montană	19	14	23	30	22	10	17	14	12	19	11	15	24	12	12	42	14	15	11, 14	28	13	13	22	35, 36	12
RM_096	Roșia Montană	16	12	21	29	19	11	19	15	11	20	10	15	22	10	11	37	17	16	12, 14	29	14	12	22	37, 38	9
RM_097	Roșia Montană	15	12	22	28	20	10	15	14	11	20	10	14	22	10	11	39	19	16	14, 14	30	13	12	26	37, 38	11
RM_099	Roșia Montană	17	13	22	30	22	9	20	13	12	20	10	16	24	10	11	41	19	14	16, 18	32	13	12	22	36, 38	13
RM_011	Roșia Montană	19	13	24	30	23	11	16	14	12	19	10	15	24	12	13	40	18	15	14, 14	32	12	12	22	35, 36	13
RM_033	Roșia Montană	19	13	21	32	21	10	17	15	11	19	11	15	24	10	11	36	18	15	14, 15	30	13	12	31	38, 39	13
RM_034	Roșia Montană	17	14	22	30	22	11	16	16	11	20	10	18	24	8	12	40	21	15	15, 15	30	14	11	26	39, 39	12
RM_101	Roșia Montană	17	13	21	31	20	9	16	13	13	20	10	17	25	9	11	39	19	14	17, 19	30	13	13	22	35, 37	12

**Table 7:** Allele frequencies and forensic parameters for 27 Y-STR loci in the Roșia Montană population. N: number of samples; Nall: number of alleles; GD: gene diversity; PIC: polymorphism information content; MP: match probability; PD: power of discrimination; STRs: short tandem repetitions

Allele/n	DYS576	DYS389I	DYS635	DYS389II	DYS627	DYS460	DYS458	DYS19	YGATAH4	DYS448	DYS391	DYS456	DYS390	DYS438	DYS392	DYS518	DYS570	DYS437	DYS449	DYS393	DYS439	DYS481	DYS533		Allele/n	DYS385		Allele/n	DYF387S1
8														0.100											11, 13	0.200		34, 34	0.033
9						0.233								0.133									0.033		11, 14	0.100		35, 36	0.367
10						0.533			0.100		0.567			0.400											12, 14	0.033		35, 36, 37	0.033
11						0.233			0.300		0.433				0.600						0.267		0.300		12, 15	0.033		35, 37	0.033
12		0.200							0.567					0.367	0.300					0.200	0.600		0.533		13, 13	0.033		36, 38	0.067
13		0.667						0.200	0.033						0.100					0.733	0.133		0.133		13, 15	0.033		37, 37	0.067
14		0.133						0.567				0.200					0.033	0.267		0.067					14, 14	0.167		37, 38	0.133
15	0.100						0.233	0.133				0.567					0.033	0.567							14, 15	0.033		37, 39	0.033
16	0.100						0.167	0.067				0.133					0.033	0.167							14, 16	0.067		37, 41	0.033
17	0.300						0.300	0.033				0.067					0.333								14, 18	0.033		38, 39	0.067
18	0.333				0.033		0.200					0.033					0.100								15, 14	0.033		39, 39	0.067
19	0.167				0.167		0.067			0.433							0.200								15, 15	0.033		39, 40	0.067
20					0.133		0.033			0.467							0.167								15, 18	0.033			
21			0.233		0.200					0.067							0.100					0.033			16, 18	0.033			
22			0.233		0.167					0.033			0.133									0.600			16, 19	0.067			
23			0.467		0.200								0.133									0.067			17, 19	0.033			
24			0.067		0.100								0.700												18, 19	0.033			
25													0.033																
26																						0.133							
28				0.167															0.067			0.067							
29				0.267															0.300			0.033							
30				0.433															0.133			0.033							
31				0.067															0.233			0.033							
32				0.033															0.200										
33				0.033															0.033										
34																			0.033										
36																0.033													
37																0.133													
38																0.100													
39																0.233													
40																0.133													
41																0.267													
42																0.100													
N	30	30	30	30	30	30	30	30	30	30	30	30	30	30	30	30	30	30	30	30	30	30	30		N	30		N	30
Nall	5	3	4	6	7	3	6	5	4	4	2	5	4	4	3	7	8	3	7	3	3	8	4		Nall	17		Nall	12
GD	0.777	0.515	0.692	0.731	0.864	0.628	0.809	0.637	0.598	0.609	0.508	0.637	0.490	0.701	0.559	0.846	0.825	0.600	0.818	0.432	0.570	0.630	0.628		GD	0.931		GD	0.848
PIC	0.711	0.445	0.613	0.662	0.814	0.539	0.748	0.573	0.511	0.503	0.371	0.573	0.437	0.618	0.466	0.793	0.772	0.513	0.760	0.370	0.485	0.586	0.541		PIC	0.893		PIC	0.806
MP	0.249	0.502	0.331	0.293	0.164	0.393	0.218	0.384	0.422	0.411	0.509	0.384	0.527	0.322	0.460	0.182	0.202	0.420	0.209	0.582	0.449	0.391	0.393		MP	0.100		MP	0.180
PD	0.751	0.498	0.669	0.707	0.836	0.607	0.782	0.616	0.578	0.589	0.491	0.616	0.473	0.678	0.540	0.818	0.798	0.580	0.791	0.418	0.551	0.609	0.607		PD	0.900		PD	0.820

**Table 8:** Haplogroups predicted for the Roșia Montană population using Athey’s haplogroup predictor by 23 Y-STRs.

ID	Haplotype	DYS393	DYS390	DYS19	DYS391	DYS385a	DYS385b	DYS439	DYS389I	DYS392	DYS389II	DYS458	DYS437	DYS448	DYS449	DYS460	YGATAH4	DYS456	DYS576	DYS570	DYS438	DYS481	DYS533	DYS635	Haplogroup	Fitness score	Probability (%)
RM_002	2	13	24	17	11	14	16	13	13	11	33	18	15	20	32	11	11	15	18	18	10	29	12	24	I2a	38	100
RM_003	3	13	22	14	10	14	0	11	12	11	28	15	15	20	28	10	11	14	16	21	10	28	11	21	I1	34	100
RM_004	4	13	24	14	11	11	13	12	13	12	29	18	15	19	29	10	12	15	18	17	12	22	12	23	R1b	36	100
RM_005	5	12	22	14	10	12	15	11	13	11	30	16	14	22	29	10	10	15	18	19	9	22	11	21	J2a	38	100
RM_006	6	12	23	15	10	14	0	11	12	11	28	15	16	20	31	10	10	14	15	20	8	26	11	22	I1	25	100
RM_007	7	13	24	14	11	11	14	11	14	13	30	17	15	19	31	11	12	15	17	17	12	22	11	23	R1b	36	100
RM_035	135	13	24	13	10	16	19	12	13	11	30	19	14	20	31	9	12	17	19	20	10	23	12	22	E1b1b	39	100
RM_043	143	13	24	14	11	11	13	12	13	12	29	18	15	19	29	10	12	15	18	17	12	22	12	23	R1b	36	100
RM_044	144	13	24	14	11	11	13	12	13	12	29	17	15	19	29	10	12	15	18	17	12	22	12	23	R1b	37	100
RM_045	145	13	24	14	11	11	14	11	14	13	30	17	15	19	31	11	12	15	17	17	12	22	11	23	R1b	36	100
RM_067	167	13	24	14	11	13	0	12	13	12	29	18	15	19	29	10	12	16	18	19	12	22	12	23	R1b	28	100
RM_070	170	12	23	15	10	14	0	11	12	11	28	15	16	20	31	10	10	14	15	20	8	26	11	22	I1	25	100
RM_074	174	12	23	13	11	14	18	12	13	11	30	18	15	21	31	10	12	14	16	17	9	22	11	23	J2a	25	100
RM_076	176	13	24	14	11	11	13	12	13	12	29	18	15	19	29	10	12	15	18	17	12	22	12	23	R1b	36	100
RM_077	177	13	24	16	10	14	15	12	13	11	30	17	15	20	32	10	11	15	17	16	10	30	13	23	I2a	46	100
RM_079	179	13	24	13	10	15	18	12	13	11	30	15	14	20	34	9	12	16	17	19	10	22	12	22	E1b1b	46	100
RM_081	181	13	24	14	11	11	13	12	13	12	29	17	15	19	29	10	12	15	18	17	12	22	12	23	R1b	37	100
RM_086	186	13	24	14	10	16	19	13	13	11	30	15	14	21	32	9	12	16	18	21	10	23	12	23	E1b1b	33	100
RM_087	187	13	24	13	10	18	19	12	13	11	31	16	14	20	32	9	12	15	17	20	10	22	12	23	E1b1b	42	100
RM_089	189	13	24	14	10	13	15	12	12	11	28	15	16	20	31	10	11	14	17	20	10	28	11	21	I1	28	100
RM_091	191	12	23	14	10	14	16	11	13	11	30	17	14	19	33	9	11	15	19	15	9	21	12	21	J2a	29	99.9
RM_092	192	13	24	14	11	11	13	12	13	12	29	17	15	19	29	11	12	15	18	17	12	22	12	23	R1b	39	100
RM_093	193	13	24	14	11	11	14	13	14	12	30	17	15	19	28	10	12	15	19	14	12	22	12	23	R1b	25	100
RM_096	196	14	22	15	10	12	14	12	12	11	29	19	16	20	29	11	11	15	16	17	10	22	9	21	G2a	40	100
RM_097	197	13	22	14	10	14	0	12	12	11	28	15	16	20	30	10	11	14	15	19	10	26	11	22	I1	37	100
RM_099	199	13	24	13	10	16	18	12	13	11	30	20	14	20	32	9	12	16	17	19	10	22	13	22	E1b1b	40	100
RM_011	211	12	24	14	10	14	0	12	13	13	30	16	15	19	32	11	12	15	19	18	12	22	13	24	R1b	22	100
RM_033	233	13	24	15	11	14	15	12	13	11	32	17	15	19	30	10	11	15	19	18	10	31	13	21	I2a	44	100
RM_034	234	14	24	16	10	15	0	11	14	12	30	16	15	20	30	11	11	18	17	21	8	26	12	22	I2b	22	100
RM_101	1101	13	25	13	10	17	19	13	13	11	31	16	14	20	30	9	13	17	17	19	9	22	12	21	E1b1b	26	100
